# Fully rubbery Schottky diode and integrated devices

**DOI:** 10.1126/sciadv.ade4284

**Published:** 2022-11-23

**Authors:** Seonmin Jang, Hyunseok Shim, Cunjiang Yu

**Affiliations:** ^1^ Department of Engineering Science and Mechanics, Pennsylvania State University, University Park, PA 16802, USA.; ^2^ Materials Science and Engineering Program, University of Houston, Houston, TX 77204, USA.; ^3^ Department of Mechanical Engineering, Texas Center for Superconductivity, University of Houston, Houston, TX 77204, USA.; ^4^ Department of Biomedical Engineering, Department of Materials Science and Engineering, Materials Research Institute, Pennsylvania State University, University Park, PA 16802, USA.

## Abstract

A fully rubbery stretchable diode, particularly entirely based on stretchy materials, is a crucial device for stretchable integrated electronics in a wide range of applications, ranging from energy to biomedical, to integrated circuits, and to robotics. However, its development has been very nascent. Here, we report a fully rubbery Schottky diode constructed all based on stretchable electronic materials, including a liquid metal cathode, a rubbery semiconductor, and a stretchable anode. The rubbery Schottky diode exhibited a forward current density of 6.99 × 10^−3^ A/cm^2^ at 5 V and a rectification ratio of 8.37 × 10^4^ at ±5 V. Stretchy rectifiers and logic gates based on the rubbery Schottky diodes were developed and could retain their electrical performance even under 30% tensile stretching. With the rubbery diodes, fully rubbery integrated electronics, including an active matrix multiplexed tactile sensor and a triboelectric nanogenerator–based power management system, are further demonstrated.

## Teaser

A stretchable Schottky diode and its integrated system are developed entirely based on soft stretchy materials.

## INTRODUCTION

The pressing needs of electronic devices that are able to mechanically deform with and morphologically conform to curvilinear and dynamic objects to address emerging challenges in health, robotics, etc. have driven the fast development of fully rubbery stretchable electronics. Among various basic electronic devices, a diode, a two-terminal electronic device with rectifying electrical properties, is ubiquitous and indispensable in widespread integrated electronic systems (*1*–*3*). A stretchable diode is a critical and indispensable device for stretchable integrated electronics toward a wide range of applications, ranging from energy (*4*–*6*), to biomedical (*7*–*9*), to integrated circuits (*10*–*12*), and to robotics (*13*–*15*). However, the development of a rubbery stretchable diode, particularly entirely based on stretchy materials, has been very nascent. Existing diodes are primarily based on conventional electronic materials of nonstretchy semiconductors and metals (*16*–*19*); thus, they are either not mechanically stretchy or have to adopt certain dedicated architecture engineering to confer structural-level macroscopic stretchability (*20*, *21*). Although a very recent effort created a stretchable, high-frequency organic diode (*22*), the major challenge toward stretchable diodes and their integrated electronics lies in the suitable energy junctions (e.g., ohmic and Schottky junctions) from rational stretchy electronic materials of conductors and semiconductors (*23*–*25*).

Here, we report a fully rubbery Schottky diode constructed all based on stretchable electronic materials, including a cathode of a eutectic liquid metal alloy of gallium-indium (liquid metal), the rubbery semiconductor of a poly(3-hexylthiophene)-nanofibril (P3HT-NF)–based composite, and an anode of a stretchable conductor of a Au nanoparticle–decorated Ag nanowire (AuNP-AgNW)–based rubber composite, which were chosen on the basis of their work functions. The rubbery Schottky diode exhibited a forward current density of 6.99 × 10^−3^ A/cm^2^ at 5 V and a rectification ratio (RR) of 8.37 × 10^4^ at ±5 V. Stretchy rectifiers and logic gates based on the rubbery Schottky diodes were developed and could retain their electrical performances even under 30% uniaxial tensile mechanical strain. Rubbery integrated electronics, including multiplexer and power management system (PMS) entirely made out of stretchy materials, are further demonstrated. An 8 × 8 arrayed rubbery multiplexed tactile sensor based on Schottky diodes was constructed and was validated to be capable of pressure mapping even when it is stretched by 30%. Furthermore, a fully rubbery PMS composed of an elastic triboelectric nanogenerator (TENG), a rubbery diode–based full-wave bridge rectifier, and a capacitor was developed and can serve as a bio-integrated self-sustained power source from organ motion. The collective results on the design and realization of the rubbery diode and integrated electronics suggest a feasible pathway toward fully rubbery integrated systems.

## RESULTS

### Fully rubbery Schottky diode

**Fig. 1.**
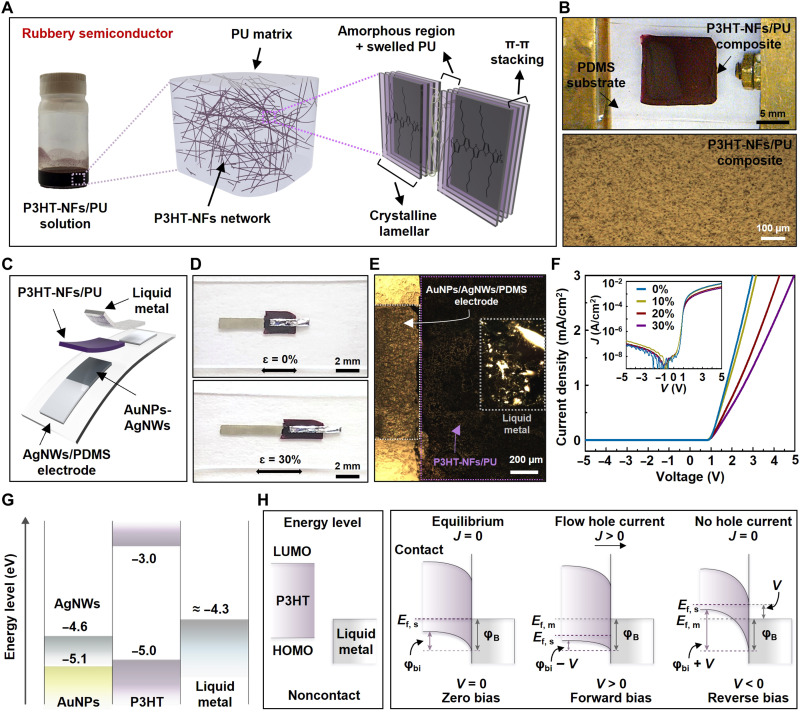
Fully rubbery Schottky diode. (**A**) An illustration of the morphology of the rubbery semiconductor composed of P3HT-NFs and PU. (**B**) An optical image and microscopic image of the P3HT-NFs/PU composite film. (**C**) Schematic illustration in an exploded view of the fully Schottky diode. (**D**) Optical images of the diode under the mechanical strain of 0 and 30%. (**E**) An optical microscopic image of the diode. (**F**) *I*-*V* characteristics of the rubbery Schottky diode under the mechanical strain of 0, 10, 20, and 30%. (**G**) Energy levels of AgNWs, AuNPs, P3HT-NFs, and liquid metal. (**H**) Illustration of the energy band diagram of the metal (liquid metal)– and p-type semiconductor (P3HT)–based Schottky junction. LUMO, lowest unoccupied molecular orbital; HOMO, highest occupied molecular orbital.

Figure 1A shows the schematic illustration of the rubbery semiconductor composite out of P3HT-NFs and polyurethane (P3HT-NFs/PU). To develop the fully rubbery semiconductor composite, P3HT dissolved in dichloromethane (DCM) was transformed into an NF structure based on low solubility–induced self-assembly at low temperature, as previously reported (*26*). Subsequently, the P3HT-NFs solution was blended with a PU solution prepared by dissolving PU in DCM to form a percolated P3HT-NFs network within the PU matrix. The preparation process of the P3HT-NFs/PU solution is demonstrated in fig. S1. The Fourier transform infrared spectroscopy (FT-IR) spectrum of the composite was examined to confirm the absorption band of the composite (fig. S2). The rubbery semiconductor shows typical FT-IR spectra of P3HT and PU with the PU-dominant spectra (see Supplementary Text and fig. S2). The optical and microscope images of the P3HT-NFs/PU composite film on a polydimethylsiloxane (PDMS) substrate are demonstrated in Fig. 1B. There were no apparent cracks or mechanical damages in the rubbery composite semiconductor film even when it was subjected to 30% tensile strain as shown in fig. S3 (A and B). The current-voltage (*I*-*V*) characteristics of the P3HT-NFs/PU composite film under the mechanical strain of 0, 10, 20, 30, and 0% (released) are presented in fig. S4A. The normalized resistance change (*R*/*R*_0_) of the film under different levels of mechanical strain is presented in fig. S4B. After releasing the applied strain, the normalized resistance was recovered from 3.183 to 1.588.

The schematic illustration in exploded view and optical images of the fully rubbery Schottky diode are illustrated in Fig. 1 (C and D, respectively). The diode consists of a AuNP-AgNW network embedded in the PDMS (AuNPs-AgNWs/PDMS), a P3HT-NFs/PU semiconductor composite, and liquid metal as the anode, p-type semiconductor, and cathode, respectively (Fig. 1E). Figure S5 shows the scanning electron microscopy (SEM) images of the P3HT-NFs, P3HT-NFs/PU, AgNWs/PDMS, and AuNPs-AgNWs/PDMS. The detailed preparations of these materials are described in Materials and Methods. The *I*-*V* characteristics of the fully rubbery Schottky diode under the mechanical strain of 0, 10, 20, and 30% are shown in Fig. 1F. The measurement was performed by sweeping the applied bias voltage from −5 to 5 V, and the RR was calculated as the ratio of the current density between −5 and 5 V. The inset presents the *I*-*V* curve, with the current density in log scale. The threshold voltage (*V*_T_) and RR of the rubbery diode are ~1 V and 8.37 × 10^4^, respectively. The rectifying characteristics of the diode remained even under the mechanical strain of 30%.

Figure 1G illustrates the energy levels of the associated electronic materials in the diode, which renders a Schottky junction between the metal and semiconductor (*27*–*29*). The AuNPs formed by galvanic replacement on the AgNWs induced a lower energy level of the electrode, thereby achieving an ohmic junction between the anode and p-type semiconducting layer (*30*, *31*). The Schottky junction between the cathode and semiconductor forms because the Fermi level of liquid metal is higher than the highest occupied molecular orbital (HOMO) level of P3HT (*29*). Different diode structures were prepared and compared to confirm the rectifying characteristic depending on different energy junctions, as shown in fig. S4 (C and D). One is a planar structure of AgNWs/PDMS-P3HT-NFs/PU-AgNWs/PDMS (Ag/P3HT/Ag) prepared by spin coating the P3HT-NFs/PU solution on the AgNWs/PDMS electrodes with a 70-μm channel. Because the Ag/P3HT/Ag structured diode has symmetric Ag-Ag electrodes, it behaved as a resistor. The other structure is AgNWs/PDMS-P3HT-NFs/PU–liquid metal (Ag/P3HT/LM). Although the Ag/P3HT/LM structured diode showed a rectifying characteristic because of the asymmetric Ag-LM electrodes, a poor rectifying characteristic (RR ≈ 25.92) was obtained because of the nonohmic contact between P3HT and Ag (*27*). These results suggest that suitable energy junctions such as Schottky and ohmic junctions between each material are crucial to achieve high rectifying characteristics. As shown in fig. S4E, the *I*-*V* characteristic of the fully rubbery Schottky diode was observed while sweeping the bias voltage from −50 to 50 V. No breakdown was observed. Figure S4F presents the electrical hysteresis of the rubbery diode with a scan rate of 3.6363 V/s. Figure 1H demonstrates the schematic energy band diagrams of the Schottky junction depending on an external bias between metal (liquid metal) and a p-type semiconductor (P3HT). When P3HT and liquid metal are contacted with zero applied bias, the electrons are transferred from liquid metal to P3HT until the Fermi levels are aligned as a consequence of thermal equilibrium. The transferred electrons remove the holes from the HOMO of P3HT, which induces an un-neutralized charge of the ionized acceptor in the P3HT to form the depletion region. In contrast, a positive charge on the metal surface is formed very thinly at atomic scale (*32*, *33*), which results in an electric field at the interface between P3HT and liquid metal, making the net current zero. The built-in potential (ϕ_bi_) is established by different work functions of P3HT and liquid metal. When the forward bias is applied, the hole carriers can flow from P3HT to liquid metal because of the low-energy barrier (ϕ_bi_ − *V*_forward_). When the reverse bias is applied, the hole carriers have to overcome a high barrier (ϕ_bi_ − *V*_reverse_). Figure S6 presents the calculated ideality factor of the rubbery Schottky diode. The value of the minimum ideality factor of the rubbery diode is approximately 1.78. We calculated the ideality factor of the rubbery diode on the basis of the thermionic emission theory (see the Supplementary Materials).

### Fully rubbery rectifiers

**Fig. 2.**
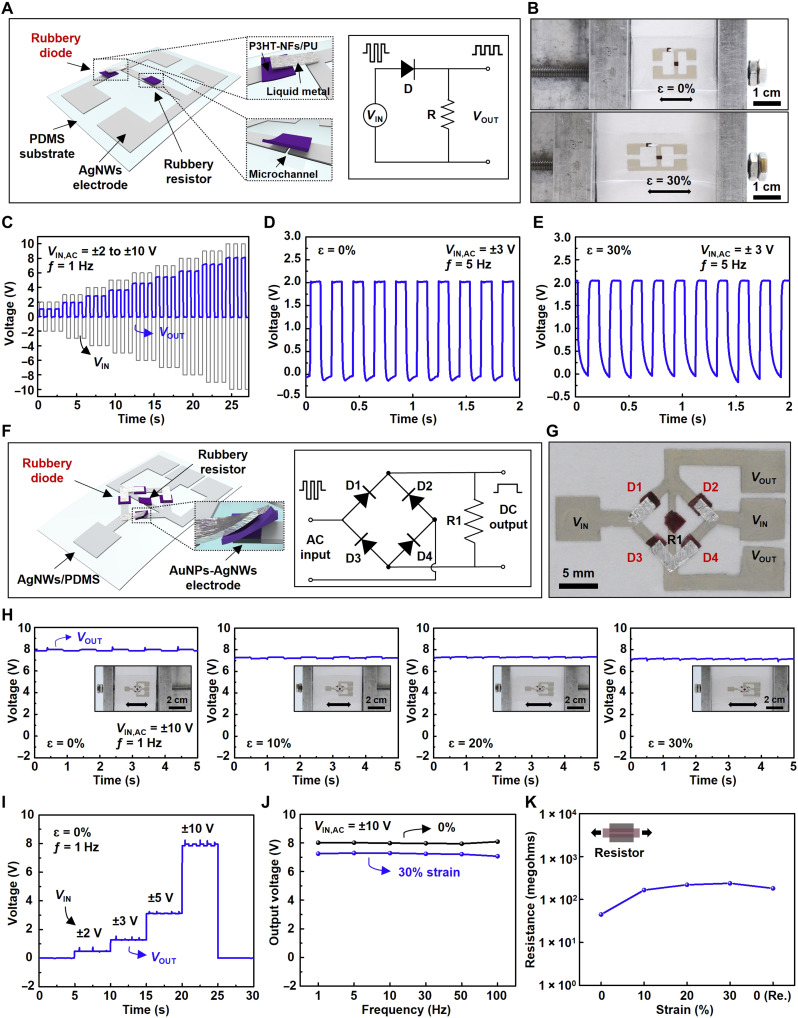
Fully rubbery rectifiers. (**A**) Schematic illustration of the half-wave rectifier and circuit diagram. (**B**) Optical images of the half-wave rectifier under the mechanical strain of 0 and 30%. (**C**) Rectified output voltages with different input voltages from ±2 to ±10 V (1 Hz). (**D** and **E**) Output voltage with *V*_IN,AC_ = ±3 V, 5 Hz under the mechanical strain of 0% (D) and 30% (E). (**F**) Schematic illustration of the rubbery full-wave bridge rectifier and circuit diagram. (**G**) An optical image of the full-wave bridge rectifier. (**H**) Output voltages with ±10 V (1 Hz) under the mechanical strain of 0, 10, 20, and 30%. (**I**) Output voltages with different input voltages of ±2, ±3, ±5, and ±10 V (1 Hz). (**J**) Frequency characteristics for the full bridge rectifier with ±10 V input voltage under the mechanical strain of 0 and 30%. (**K**) Resistance of the rubbery resistor under the mechanical strain of 0, 10, 20, 30, and 0% (released) perpendicular to the channel length.

A rectifier is an important electronic component used for power conversion (*34*), transformer (*35*), and voltage multiplier (*36*). Figure 2A presents the schematic illustration of a fully rubbery half-wave rectifier, which is composed of a rubbery diode and a load resistor. The rubbery load resistor was prepared by spin coating the P3HT-NFs/PU composite onto the AgNWs/PDMS electrodes to form a two-terminal resistive device. Figure 2B shows the optical images of the half-wave rectifier under 0 and 30% tensile strain. The rectification performance of a fully rubbery half-wave rectifier is shown in Fig. 2C. Rectified output voltages (blue curve) were obtained, while various pulses (peak-to-peak; gray curve) ranging from **±**2 to **±**10 V were applied with a pulse frequency of 1 Hz and 50% duty cycle. The threshold voltage of the rubbery diode is responsible for the reduction in output voltage relative to the input voltage. Figure 2 (D and E) represents the rectified output voltages of the rubbery rectifier without and with 30% mechanical strain when the input voltage of ±3 V (pulse frequency: 5 Hz; 50% duty cycle) was applied. These results show that the rectifying characteristic of the rubbery Schottky diode is retained even when the device is mechanically stretched by 30%.

A full-wave bridge rectifier is a core component in energy harvesters, such as piezoelectric or triboelectric generators, to convert AC into DC (*37*, *38*). A rubbery full-wave bridge rectifier composed of four diodes and one resistor is schematically depicted in Fig. 2F. Figure 2G shows an optical image of the rubbery full-wave bridge rectifier. A set of optical images of the full-wave bridge rectifier under the mechanical stretching of 0, 10, 20, and 30% is shown in Fig. 2H. Unlike the half-wave rectifier, the full-wave bridge rectifier maintains a positive voltage when a negative input voltage is applied, as opposed to decreasing to 0 V. The voltage drop from 10 to 8 V was due to the current pathway through two diodes in the full-wave bridge rectifier. Upon stretching by 30% strain, the rectifying characteristic of the full-wave bridge rectifier was maintained, and the output voltage did not show a notable decrease. Figure 2I presents the rectifying characteristics of the rubbery full-wave bridge rectifier corresponding to various input voltages, ranging from ±2 to ±10 V. Figure 2J exhibits the rectifying characteristics dependent on the input frequency of the rectifier with and without 30% mechanical strain. Both output voltages under the mechanical strain of 0 and 30% were maintained without significant output voltage drop when the frequency increased from 1 to 100 Hz. Note that the direction of the applied mechanical strain to the load resistor is perpendicular to the channel length and the resistance of the load resistor increased, as shown in Fig. 2K. However, because the resistance of the diodes also increased as the device is stretched, no marked output voltage change was observed. The fabrication processes of the half-wave and full-wave bridge rectifiers are shown in fig. S7 (A and B). Such a fully rubbery full-wave bridge rectifier could be integrated with an energy harvester for applications where large mechanical deformations are associated with, as illustrated in the following.

### Rubbery logic gates

**Fig. 3.**
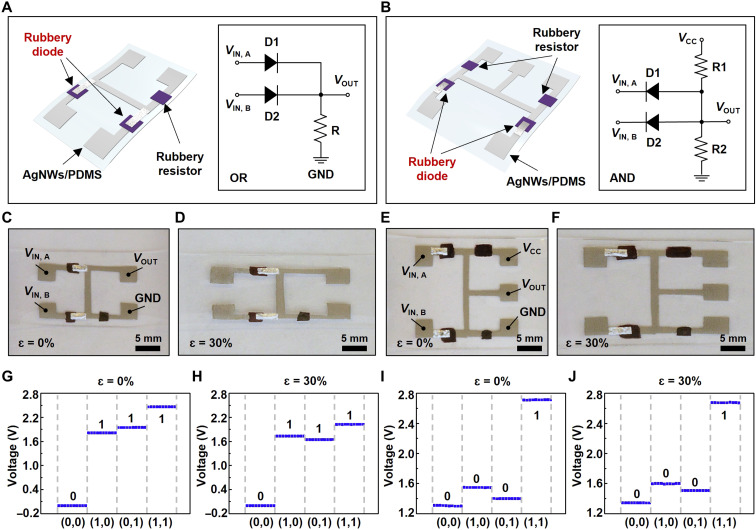
Rubbery logic gates. (**A** and **B**) Schematic illustrations of the rubbery OR gate (A) and AND gate (B). (**C** and **D**) Optical images of the rubbery OR gate under the mechanical strain of 0% (C) and 30% (D). (**E** and **F**) Optical images of the rubbery AND gate under the mechanical strain of 0% (E) and 30% (F). (**G** and **H**) Output characteristics of rubbery OR gate under the mechanical strain of 0% (G) and 30% (H). (**I** and **J**) Output characteristics of rubbery AND gate under the mechanical strain of 0% (I) and 30% (J). GND is short for ground.

Logic gates implementing Boolean operations in digital systems are the fundamental building blocks of digital integrated electronic circuits (*39*). Here, we developed fully rubbery logic gates of OR and AND based on the rubbery Schottky diodes. Figure 3 (A and B) presents the schematic illustrations of the rubbery OR and AND logic gates, and the insets show the corresponding circuit diagrams. The anodes of the diodes serve as the input electrodes for the rubbery OR gate. In contrast, the cathodes of the diodes serve as input electrodes in the rubbery AND gate. In the case of the rubbery AND gate, an external input voltage (*V*_CC_) is applied via a resistor (R1) to the anodes of diodes to produce a forward-biased diode when no input voltages are present at the initial state. The logic function characteristics of the rubbery logic gates were examined by measuring the output voltages while applying four combinations of input voltages (*V*_IN,A_,*V*_IN,B_) that correspond to (0,0), (1,0), (0,1), and (1,1) states. Logic states of 0 and 1 for both *V*_IN,A_ and *V*_IN,B_ are based on the input voltages of 0 or 3 V, respectively. Figure 3 (C and D) shows the optical images of the rubbery OR gate under the mechanical strain of 0 and 30% (along the channel length of the resistor). Figure 3 (E and F) shows the optical images of the rubbery AND under the mechanical strain of 0 and 30% (along the channel length of the resistor). The output voltages (*V*_OUT_) of the rubbery OR gate depending on different input voltages are plotted in Fig. 3G. The results indicate that the logic state 0 of the OR gate can be achieved only when both inputs (*V*_IN,A_ and *V*_IN,B_) are at logic state 0. Figure 3H shows that the rubbery OR gate retained its logic function under the mechanical strain of 30%. Figure 3 (I and J) presents the *V*_OUT_ of the rubbery AND gate without and with 30% strain. The AND gate, in contrast, only displays logic state 1 when both inputs are logic state 1. The truth tables of both the OR and AND gates are presented in fig. S8. These results validate that fully rubbery logic gates can be operated without disturbing their logic functions under the mechanical strain of 30%. The fabrication processes of the rubbery logic gates are shown in fig. S7 (C and D).

### Fully rubbery multiplexed tactile sensor array

**Fig. 4.**
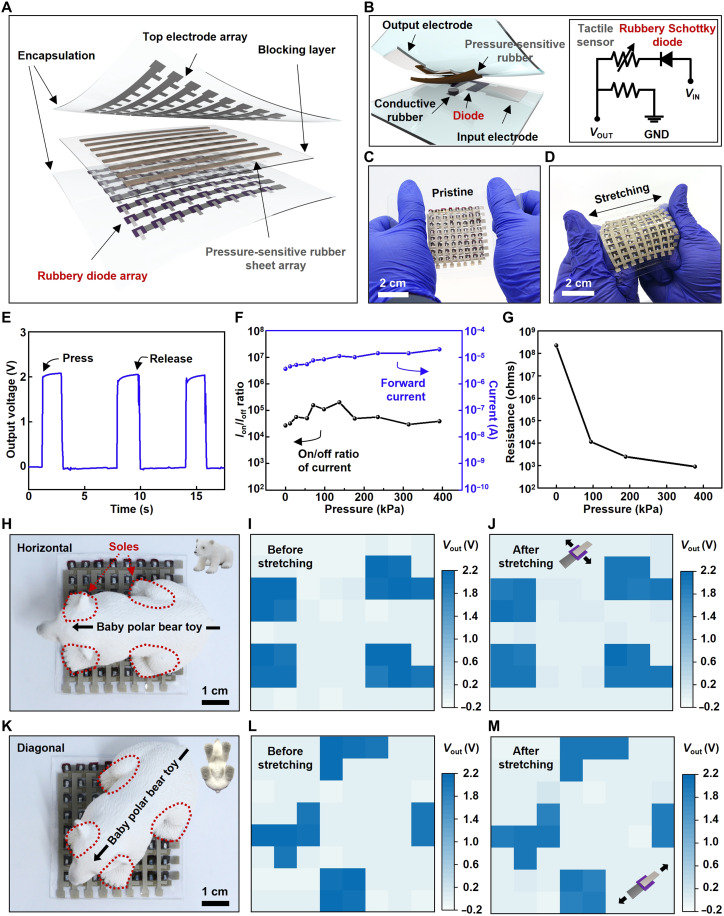
Fully rubbery multiplexed tactile sensor array. (**A**) Schematic illustration in an exploded view of the fully rubbery multiplexed tactile sensor array. (**B**) Schematic illustration in an exploded view and circuit diagram of the single-sensing pixel. (**C** and **D**) The optical images of the fully rubbery multiplexed tactile sensor array without (C) and with stretching (D). (**E**) Output characteristic of the tactile-sensing pixel in the matrix with and without external pressure. (**F**) RR and forward current of the fully rubbery Schottky diode at 5 V under different pressures. (**G**) Dependence of the resistance of the pressure-sensitive rubber sheet on applied pressure. (**H**) Optical images of the fully rubbery multiplexed tactile sensor array with the toy (horizontal). (**I** and **J**) Output voltage mapping before (I) and after (J) stretching along the top electrode array. (**K**) Optical images of the fully rubbery multiplexed tactile sensor array with the toy (diagonal). (**L** and **M**) Output voltage mapping before (L) and after (M) stretching along the bottom electrode array.

We developed an 8 × 8 arrayed, rubbery multiplexed tactile sensor based on the rubbery Schottky diodes. The diodes serve as the active multiplexing units, which suppress the cross-talk effect during spatiotemporal tactile mapping because of its rectifying characteristics (*40*). The fully rubbery tactile sensor array was constructed with 8 × 8 sensing nodes consisting of 64 diodes (bottom layer), eight lines of the pressure-sensitive rubber sheet (middle layer), and eight lines of the rubbery electrode (top layer). The schematic illustration of the sensor device and also a single pixel in an exploded view is shown in Fig. 4 (A and B). The inset in Fig. 4B shows the circuit diagram of a single pixel. A cross-sectional microscopic image of the rubbery tactile sensor is shown in fig. S9. The circuit diagram of the 8 × 8 arrayed active matrix multiplexed tactile sensor and the detailed fabrication process are demonstrated in figs. S10 and S11, respectively. Figure 4 (C and D) shows the optical images of the rubbery tactile sensor array with and without stretching. Figure 4E shows a measured output voltage from cyclic pressing and releasing on a single pixel while applying 3 V. The voltage drop mainly resulted from the threshold voltage of the rubbery diode. The cyclic pressing and releasing test on the pressure-sensitive rubber sheet without the diode was examined as shown in fig. S12. Figure 4F demonstrates the rectifying characteristics of the rubbery diode under different pressure ranging from 0 to 400 kPa. The result reveals that the rubbery diode’s on/off current ratio (*I*_on_/*I*_off_) was maintained above 10^4^ even under external pressure. Figure 4G shows the resistance change of the pressure-sensitive rubber sheet depending on applied pressure.

While an object (a polar bear toy) was placed on the rubbery tactile sensor array, the resistance of the pressure-sensitive rubber sheet decreased. Thus, the voltage change across the resistor connected to the pressure-sensitive rubbery sheet can be detected. Once the applied pressure was higher than 50 kPa, each pixel was activated with the applied voltage of 3 V. Figure 4H shows the horizontally placed toy on the rubbery tactile sensor array. The pressure was applied to the top surface by the four soles of the toy (red dotted lines). The map of output voltage obtained from each pixel depending on the applied pressure is shown in Fig. 4I. After the stretching by 30% along the top electrode array, the map of output voltage was obtained without substantial change, as presented in Fig. 4J. Figure 4K shows the diagonally placed toy on the rubbery tactile sensor array. The map of output voltage from each pressed pixel before and after 30% of mechanical strain along the bottom electrode array (orthogonal to the top electrode array) can be obtained as shown in Fig. 4 (L and M, respectively). As a comparison, a tactile sensor array without the diodes was fabricated to confirm the cross-talk effect compared with the multiplexed tactile sensor array, as presented in fig. S13. The results show that undesired output signals from nonpressed pixels were detected when an object was placed on the tactile sensor array without the rubbery multiplexing diodes. Such tactile sensor could be used in various emerging applications such as wearable sensors, electronic skin, and soft robotics.

### Fully rubbery PMS

**Fig. 5.**
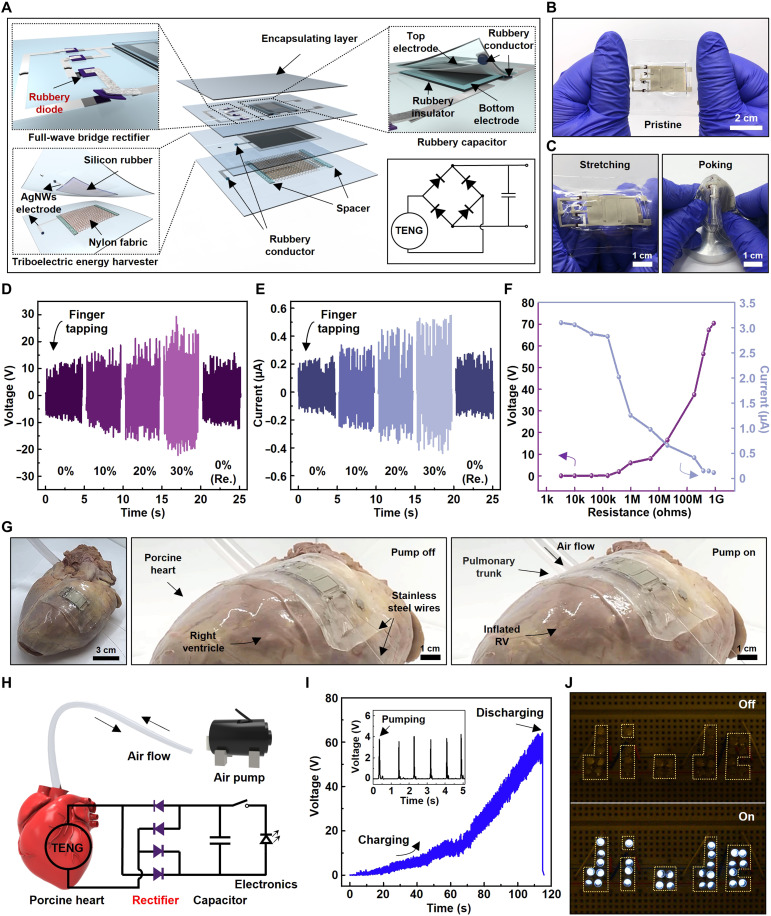
Fully rubbery PMS. (**A**) Schematic illustration in an exploded view and circuit diagram of the fully rubbery PMS. (**B** and **C**) Optical images of the fully rubbery PMS without (B) and with mechanical deformation (C). (**D** and **E**) Output voltage (D) and current (E) of the nylon/PDMS-based TENG with finger tapping under the mechanical strain of 0, 10, 20, 30, and 0% (released). (**F**) Output voltage and current of the TENG dependent on external resistors. (**G**) Optical images of a laboratory-made heart pumping system made of the dead porcine heart. (**H**) Schematic diagram of the power-generating system. (**I**) A measured voltage of the rubbery PMS during pumping of the porcine heart. (**J**) Photograph of 29 light-emitting diodes powered by the fully rubbery PMS.

We further demonstrated a fully rubbery PMS consisting of a rubbery TENG, a full-wave bridge rectifier, and a capacitor to highlight the potential of the fully rubbery Schottky diode for a deformable energy harvester, which is desired in implantable biomedical devices (*41*, *42*). However, these existing energy-harvesting systems are exclusively based on rigid diodes (*43*, *44*). The detailed fabrication process of the PMS is described in Materials and Methods. Figure 5A shows the schematic illustration in an exploded view of the fully rubbery PMS. To construct the rubbery TENG, a stretchy nylon fabric that has weaker electron affinity was selected as a bottom layer of the TENG, and a thin PDMS film that has stronger electron affinity served as a top layer (*45*–*47*). The detailed fabrication process and working mechanism of the nylon/PDMS-based TENG driven in a contact separation mode are depicted in figs. S14 and S15, respectively. The optical image of the fully rubbery PMS is presented in Fig. 5B. The rubbery PMS is mechanically deformable under various modes, such as stretching and poking, as shown in Fig. 5C.

The electrical performances of the rubbery TENG under the tensile strain of 0, 10, 20, and 30% were characterized by finger tapping. As the strain increased, the output voltage and current increased because of the increased surface area in contact separation mode, as shown in Fig. 5 (D and E) (*48*). Upon releasing the strain, both voltage and current recovered to the original values. Note that the energy-harvesting performance remained even after 100 cycles of stretching and releasing. Figure 5F shows the dependence of both voltage and current outputs of the TENG with different external load resistors. The output power density depending on external load resistors is shown in fig. S16.

To illustrate its usage as a soft deformable implantable energy harvester, we prepared a porcine heart, which was pumped by an external air pump to imitate the rhythmic contraction of the cardiac cycle. Figure 5G shows the optical images of a porcine heart with a rubbery PMS stick on the epicardial surface. A plastic tube was stuck in the pulmonary trunk to blow air into the right ventricle, mimicking a cardiac cycle. Figure 5H depicts the schematic diagram of the experimental setup as well as the electronic circuit of the PMS. The rubbery PMS applied to the surface of the right ventricle of the porcine heart generated bidirectional voltage when the heart was periodically pumped. Subsequently, the voltage was rectified via the rubbery full-wave bridge rectifier, and then the electrical charge was stored in the rubbery capacitor (~80 pF). The schematic illustration in an exploded view and fabrication process of the rubbery capacitor is illustrated in fig. S17. Capacitances dependent on the thickness of the rubber capacitor were investigated, as shown in fig. S18. Figure 5I demonstrates the charging and discharging curves of the rubbery PMS. After charging for ~2 min, the rubbery PMS started to power 29 light-emitting diodes (LEDs) arranged in the letters of “diode,” as shown in Fig. 5J. Note that 29 LEDs were connected in series as illustrated in fig. S19. The results show that a fully rubbery PMS is potentially applicable to power various medical implants, such as a pacemaker.

## DISCUSSION

We have reported the fully rubbery Schottky diode and various functional components and integrated electronics systems. Constructing Schottky diodes fully based on stretchy materials, including two different kinds of metals, semiconductors, and substrate, suggests a simple and feasible way toward stretchable diodes and electronics, which bypasses the needs of any dedicated architecture engineering based on conventional rigid, nonstretchy electronic materials (*49*–*51*). A relatively high RR of the rubbery diode and robust device performance under high level tensile strain indicate rational device design and render its applications in various integrated systems, including the arrayed rubbery tactile sensor and the rubbery PMS-based epicardial energy harvester. The rubbery diodes and their integrated electronics and systems hold promise in a wide range of applications, including but not limited to bioelectronics (*43*), to smart skins (*52*), to soft robotics (*53*), and to humanoids (*54*), among others.

## MATERIALS AND METHODS

### Materials

Anhydrous DCM (>99.8%), acetone (>99.9%), isopropyl alcohol (IPA; >99.5%), PU pellets, regioregular P3HT, liquid metal (eutectic gallium-indium), gold chloride trihydrate (HAuCl_4_·3H_2_O; >99.9%), and anhydrous ammonia (NH_4_OH; 28%) were all purchased from Sigma-Aldrich and used as received. The PDMS silicone elastomer kit (Sylgard 184) was from Dow Corning. AgNW solution (1 weight % in IPA; average diameter, 90 nm; length, 40 μm) was purchased from Zhejiang Kechuang Advanced Materials. Pressure-sensitive rubber sheets (ZL45.1) and conductive rubber pastes (FL45) were from Zoflex.

### Preparation of partially galvanic exchanged AuNPs-AgNWs/PDMS electrode

To obtain the stretchable AuNPs-AgNWs/PDMS electrode, an AgNW solution was drop-cast onto a clean glass substrate with a shadow mask prepared by a programmable cutting machine (Silhouette Cameo) and then baked at 80°C for 10 min. A mixture of PDMS elastomer [10:1 (w/w) prepolymer/curing agent] (Sylgard 184, Dow Corning) was spin-coated onto the patterned AgNW electrode at 300 rpm for 60 s, and then the sample was cured at 80°C for 120 min in a dry oven. Because of a higher density of AgNWs at the bottom than at the top and the high viscosity of the PDMS mixture, a partially embedded AgNWs/PDMS electrode was obtained (*55*). The preparation of the AgNWs/PDMS composite electrode was completed by peeling off the cured PDMS from the glass substrate. An aqueous solution of 0.5 mM HAuCl_4_ was dropped onto desired areas on the AgNWs/PDMS electrode to form AuNPs on AgNWs through the galvanic exchange process for 15 min (*30*, *31*). The treated AuNPs-AgNWs composite was cleaned with deionized (DI) water, followed by immersing NH_4_OH solution (28%) for 5 min to remove the by-product of AgCl. Last, the stretchable AuNPs-AgNWs/PDMS electrode was completed by cleaning with DI water, drying with an N_2_ gun, and dehydrating at 120°C for 2 min on a hot plate.

### Preparation of the P3HT-NFs/PU composite

The P3HT powder was dissolved in DCM (6 mg/ml) at 80°C and then cooled down to −20°C for 30 min. The cooling down process in the P3HT solution led to a conformational change of the P3HT chain to NFs (*56*). Separately, the PU pellet was dissolved in DCM (100 mg/ml) at 80°C. The P3HT-NFs solution was blended with the PU solution to form the P3HT-NFs/PU composite at a weight ratio of 1:2.7. The prepared solution was kept at −20°C.

### Fabrication of the rubbery diode, rectifiers, and OR and AND logic gates

The fully rubbery diode was fabricated by first preparing the AuNPs-AgNWs/PDMS electrode. After sonicating the pre-prepared P3HT-NFs/PU composite solution for 30 s, the P3HT-NFs/PU semiconductor was patterned by spin coating on the AuNPs-AgNWs/PDMS at 1000 rpm for 30 s through a Kapton film–based shadow mask and annealed at 110°C for 30 min. Next, the liquid metal electrode was patterned on the P3HT-NFs/PU through a Kapton film shadow mask. Last, the rubbery resistor was fabricated by spin coating the P3HT-NFs/PU solution at 1000 rpm for 30 s through a shadow mask on two electrodes with a gap of 80 μm, followed by annealing at 110°C for 30 min.

### Fabrication of fully rubbery multiplexed tactile sensor array

The fabrication of the rubbery Schottky diode array as the bottom layer followed the same process as described above. Next, a PDMS layer with holes was laminated on the diode array as the blocking layer. The blocking layer was prepared to prevent direct contact between the top and bottom electrodes, and the holes were used to connect a pressure-sensitive rubber sheet and liquid metal. The pressure-sensitive rubber sheet array was aligned on the holes of the blocking layer, and then the holes were filled with the conductive rubber paste, followed by heating on the hot plate at 50°C for 8 hours. Last, the top electrode layer was placed on the pressure-sensitive rubber sheet array with the electrode side facing down, and then the edges of the whole layer were sealed with the PDMS solution and then cured at 80°C for 60 min.

### Fabrication of fully rubbery PMS

The rubbery PMS was constructed with two main parts: (i) TENG and (ii) full-wave bridge rectifier and capacitor. The fabrication of the rubbery PMS started with creating the rubbery TENG. Two AgNWs/PDMS-based electrodes were prepared for the bottom and top layers of the TENG, with the same process of AgNWs/PDMS electrode preparation as described above. To prepare the nylon/PDMS-based rubbery TENG, a piece of nylon fabric obtained from women’s pantyhose (100% nylon, No Nonsense) was layered on the AgNWs/PDMS electrode and then fixed with a PDMS solution and cured on the hot plate at 80°C for 60 min (nylon/AgNWs/PDMS as the bottom layer of the TENG). The PDMS solution was spin-coated on the AgNWs/PDMS electrode at 3000 rpm for 60 s through a Kapton film–based shadow mask and cured for 30 min at 110°C (thin PDMS/AgNWs/PDMS as the top layer of the TENG). Two PDMS spacers (thickness, 1.2 mm) were placed on the edge of the nylon piece of the bottom layer in parallel to maintain separation between the top and bottom layers. To assemble the top and bottom layers of the TENG, the top layer was placed on the PDMS spacers with the thin PDMS layer facing down. After finishing the fabrication of the rubbery TENG, the via holes were created to allow the interconnection between the TENG and the rubbery full-wave bridge rectifier. Next, the rubbery rectifier and capacitor were prepared. First, AgNW electrodes for both the rectifier and capacitor were patterned on the same PDMS substrate with a Kapton film–based shadow mask. To prepare a PDMS-based rubbery capacitor, a PDMS solution was spin-coated on the bottom electrode of the capacitor masked by a Kapton film at 3000 rpm for 30 s, and then the pre-prepared AgNWs/PDMS top electrode for the capacitor was placed on the surface of the uncured PDMS with the electrode side facing down, followed by curing at 110°C for 10 min. The conductive rubber paste was used to connect the capacitor to the PMS circuit electrically. Next, four diodes were fabricated on the pre-patterned AgNW electrode on the PDMS substrate to create the rubbery full-wave bridge rectifier using the same process described above, and then via holes were created on the rectifier for connection between the TENG and the rectifier. The PDMS substrate containing the rectifier and capacitor was placed on top of the TENG to integrate the rubbery TENG, rectifier, and capacitor while aligning the via holes of the TENG and rectifier layers. After that, the via holes were filled with conductive rubber paste and then solidified at 50°C for 8 hours. Last, the PDMS solution was spin-coated at 250 rpm for 60 s to encapsulate the device and then cured on the hot plate at 80°C for 2 hours.

### Material characterization and device measurements

The electrical characterization of the diode and resistor was performed by a semiconductor analyzer (4200SCS, Keithley Instruments Inc.). A function generator (DG4062, RIGOL Technologies Inc.) and DC power supply (1627A, BK Precision) were used to characterize the rectifiers and the OR and AND logic gates. The output voltages were measured by a digital multimeter (DMM6500 6 ½, Keithley Instruments Inc.). The output voltage and current of the TENG were measured by an electrometer (6514, Keithley Instrument Inc.). The absorption bands of P3HT-NFs, PU, and P3HT-NFs/PU composite were measured using an FT-IR spectrometer (Nicolet iS50, Thermo Fisher Scientific). The surface morphologies of the P3HT-NFs, P3HT-NFs/PU composite, AgNWs, and AuNPs-AgNWs were investigated by SEM (LEO 1525, Zeiss).

## Supplementary Materials

### This PDF file includes:

Supplementary Texts Figs. S1 to S19 References
