# Supplementary material for: Fully rubbery Schottky diode and integrated devices
Source: Sci Adv. 2022 Nov 23;8(47):eade4284. doi: 10.1126/sciadv.ade4284 (PMC9683705; doi:10.1126/sciadv.ade4284)
Supplement: Supplementary file 1 — Supplementary Texts Figs. S1 to S19 References [file sciadv.ade4284_sm.pdf]

Science Advances
AAAS

Supplementary Materials for

Fully rubbery Schottky diode and integrated devices

Seonmin Jang *et al.*

Corresponding author: Cunjiang Yu, cmy5358@psu.edu

Sci. Adv. **8**, eade4284 (2022)
DOI: 10.1126/sciadv.ade4284

This PDF file includes:

Supplementary Texts
Figs. S1 to S19
References

Supplementary Texts

Analysis of FT-IR measurement

Representative absorption peaks of P3HT-NFs semiconducting polymer are shown at 2957, 2924 and 2851 cm^{-1} which are attributed to C-H aliphatic stretch (stretching). The peaks of thiophene ring appear at 1485 (stretching) and 1377 cm^{-1} (methyl deformation). The C-C vibration are observed at 1260 cm^{-1}. Strong absorption peak of the aromatic C-H stretching is emerged at around 800 cm^{-1}, as reported (*57*). For absorption band of PU, the peak of the N-H stretching appear at 3329 cm^{-1} while the C-H stretching peaks are shown at 2934 and 2850 cm^{-1}. The absorption band at 1716 cm^{-1} is attributed to C=O carbonyl stretching. The δN-H with CO-N vibration and C-N vibration with δN-H are shown at a1526 cm^{-1} and 1230 cm^{-1}, respectively (*58*). To compare a chemical bonding variation after blending P3HT-NFs and PU, spectra of the P3HT-NFs/PU composite also was investigated. As shown in fig. S2, the absorption bands of PU dominate on the FT-IR spectrum due to the relatively high amount of PU in the composite. The intensity of the peak at around 801 cm^{-1} increases on the mixed P3HT-NFs/PU composite than the PU spectrum.

Ideality factor Calculation

Ideality factors can be estimated from the slope of a plot of log I versus V plot (*59, 60*). The ideality factor of the rubbery Shottky diode is calculated using

$$\boldsymbol{n} = \left(\frac{\boldsymbol{q}}{\boldsymbol{kT}}\frac{\boldsymbol{dV}}{\boldsymbol{d(lnJ)}}\right) = \left(\frac{\boldsymbol{kT}}{\boldsymbol{q}}\frac{\boldsymbol{d(lnJ)}}{\boldsymbol{dV}}\right)^{-1}$$

Where, n is the ideality factor, q is the elementary electric charge, k is the Boltzmann constant, T is the absolute temperature, V is the applied voltage, and J is the current density through the diode.

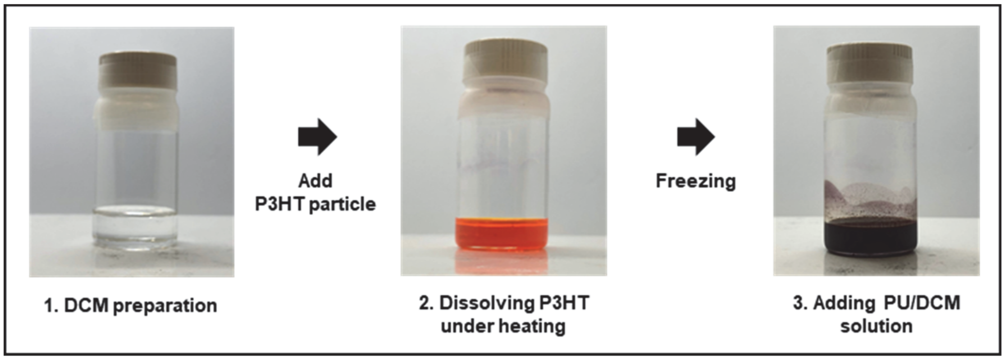

Fig. S1. The preparation process of the P3HT-NFs/PU solution.

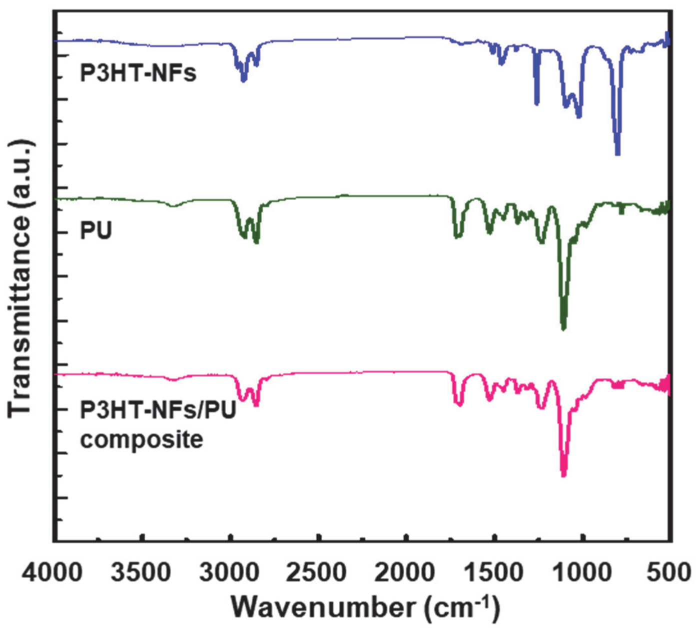

Fig. S2. FT-IR spectra of the P3HT-NFs, PU and P3HT-NFs/PU composite.

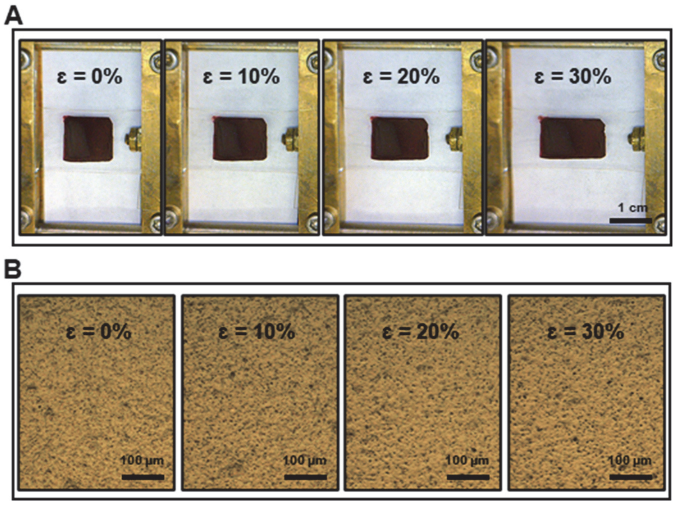

Fig. S3. The P3HT-NFs/PU rubbery semiconductor composite film under mechanical strain of 0, 10, 20 and 30%. (**A**) Optical images. (**B**) Optical microscopic images.

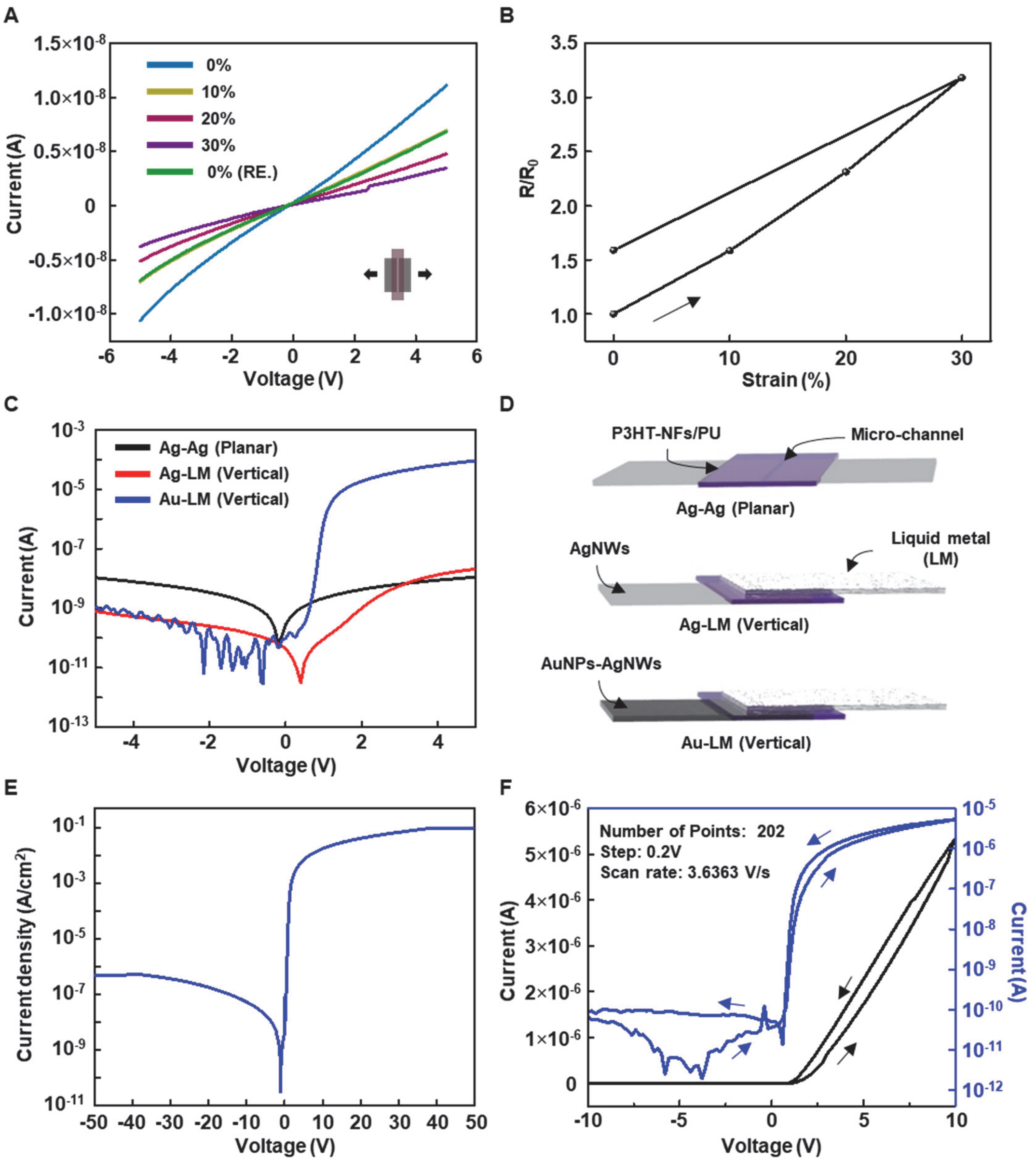

Fig. S4. Electrical properties of the rubbery Schottky diodes with different device structures. (**A**) I-V characteristics of P3HT-NFs/PU composite film on micro-channel under mechanical strain of 0, 10, 20, 30 and 0% (released) perpendicular to the channel length. (**B**) Relative resistance change under strain. (**C**) I-V characteristics of the rubbery diodes with different structures and metal-semiconductor junctions. (**D**) Schematic illustration of three different structures. (**E**) Rectifying property of the rubbery diode under -50 to 50 V voltage sweep. (**F**) I-V hysteresis of the diode with scan rate of 3.6363 V/s.

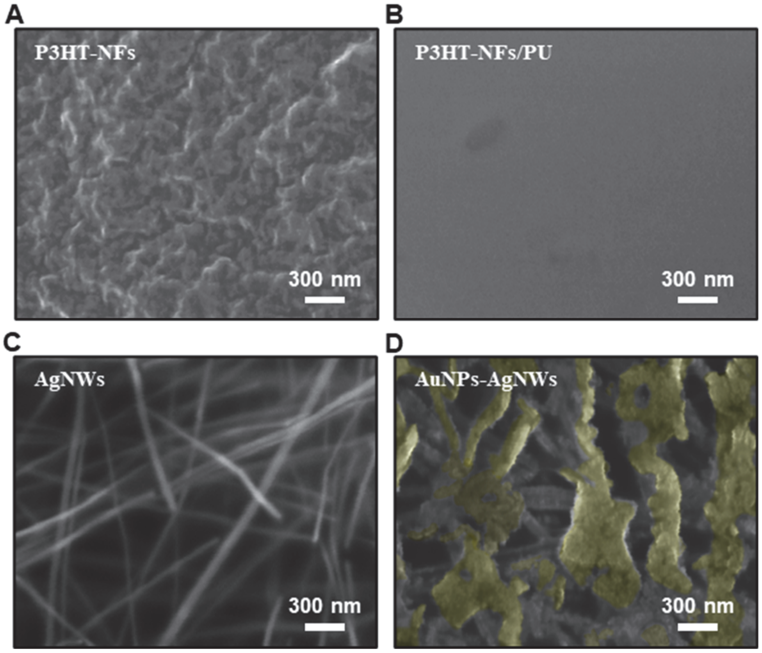

Fig. S5. SEM images of semiconductors and conductors. (**A**) P3HT-NFs. (**B**) P3HT-NFs/PU. (**C**) AgNWs. (**D**) AuNPs-AgNWs.

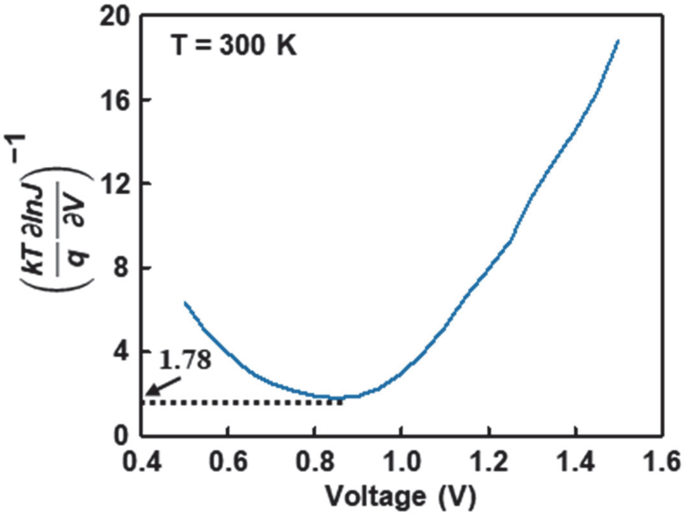

Fig. S6. Calculated ideality factor of the rubbery Schottky diode.

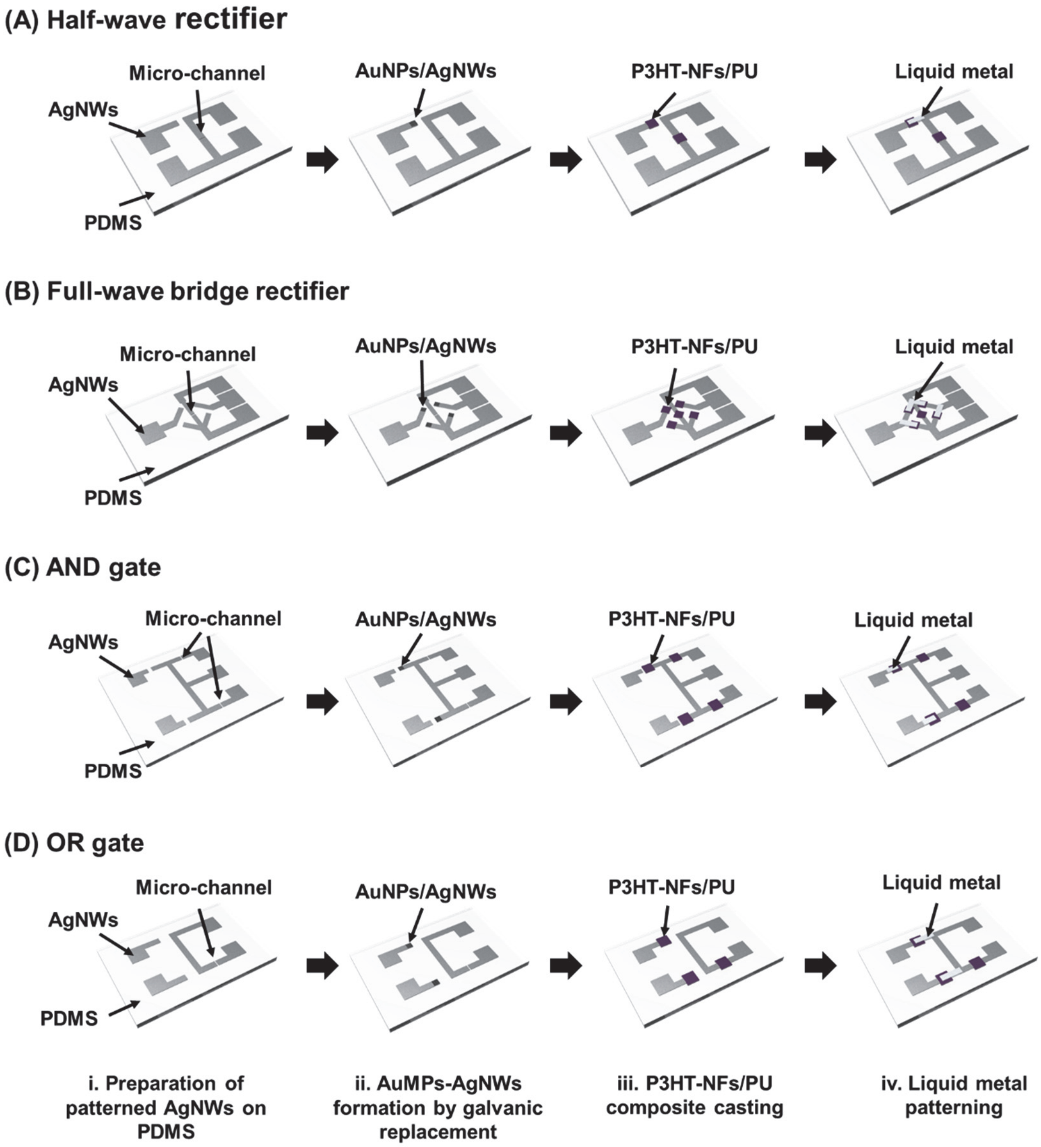

Fig. S7. Schematic illustration of the fabrication process of the rubbery rectifiers and logic gates. (**A**) Half-wave rectifier. (**B**) Full-wave bridge rectifier. (**C**) AND gate. (**D**) OR gate.

A

Rubbery OR gate

$V_{IN, A}$	$V_{IN, B}$	V_{OUT} (0% strain)	V_{OUT} (30% strain)
0	0	0	0
1	0	1	1
0	1	1	1
1	1	1	1

B

Rubbery AND gate

$V_{IN, A}$	$V_{IN, B}$	V_{OUT} (0% strain)	V_{OUT} (30% strain)
0	0	0	0
1	0	0	0
0	1	0	0
1	1	1	1

Fig. S8. Truth tables of the rubbery logic gates. (**A**) OR gate. (**B**) AND logic gate.

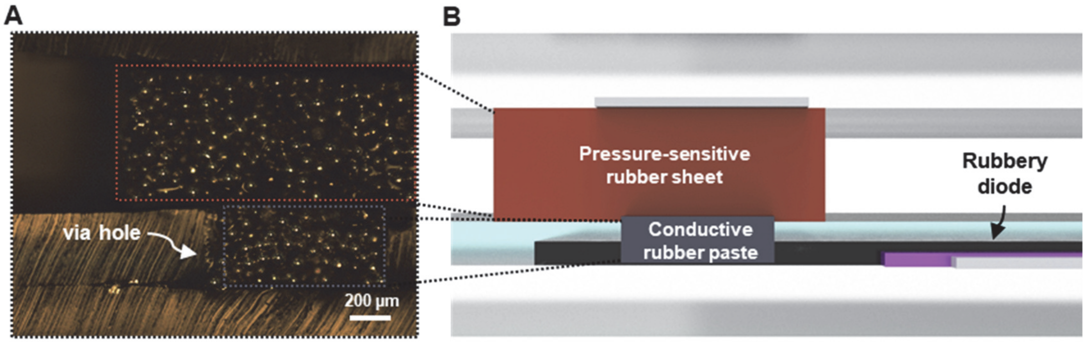

Fig. S9. The rubbery tactile sensor. (**A**) A cross-sectional optical microscopic image. (**B**) A cross-sectional schematic illustration of the tactile sensor

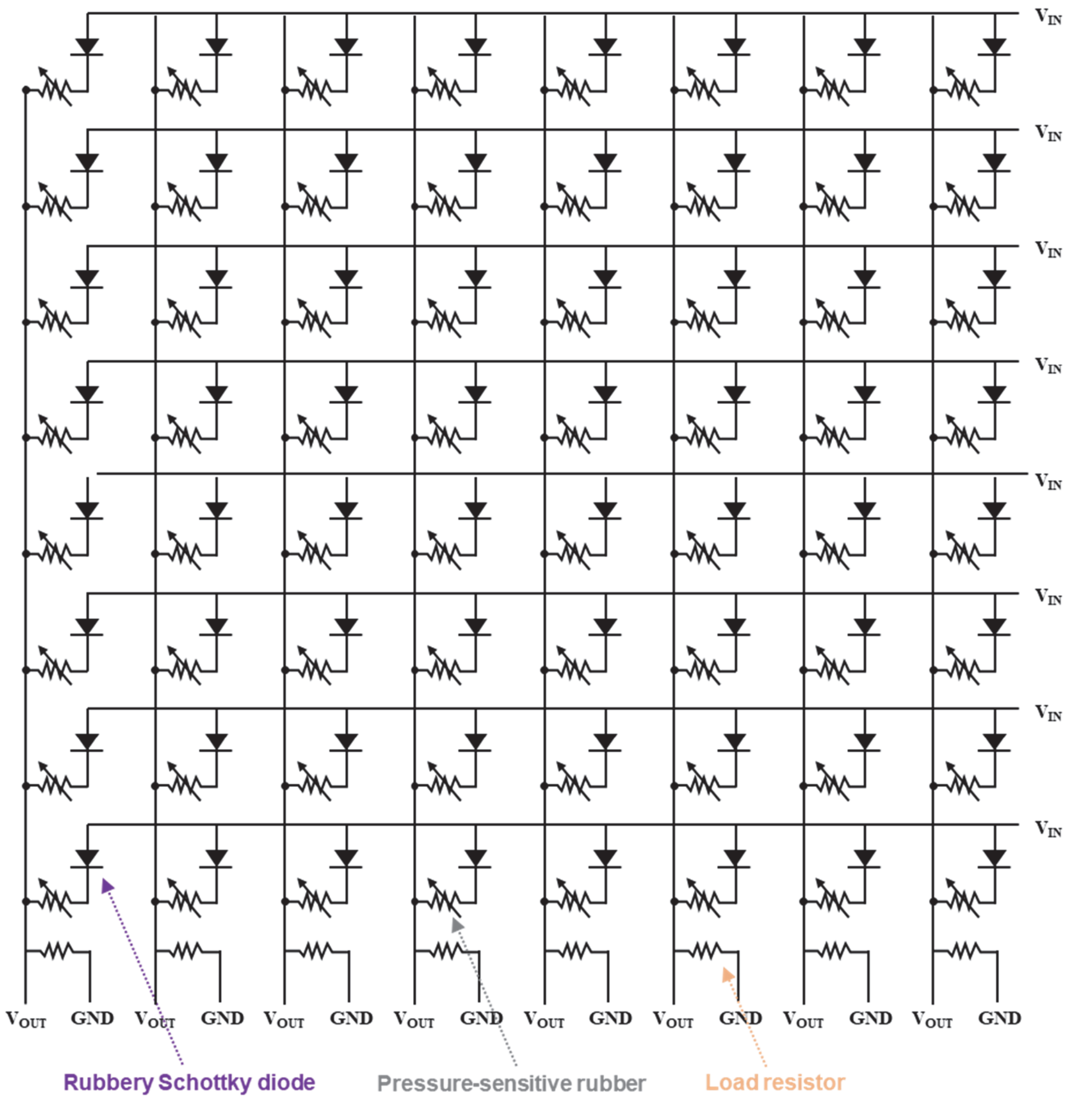

Fig. S10. Circuit diagram of the 8×8 arrayed rubbery multiplexed tactile sensor.

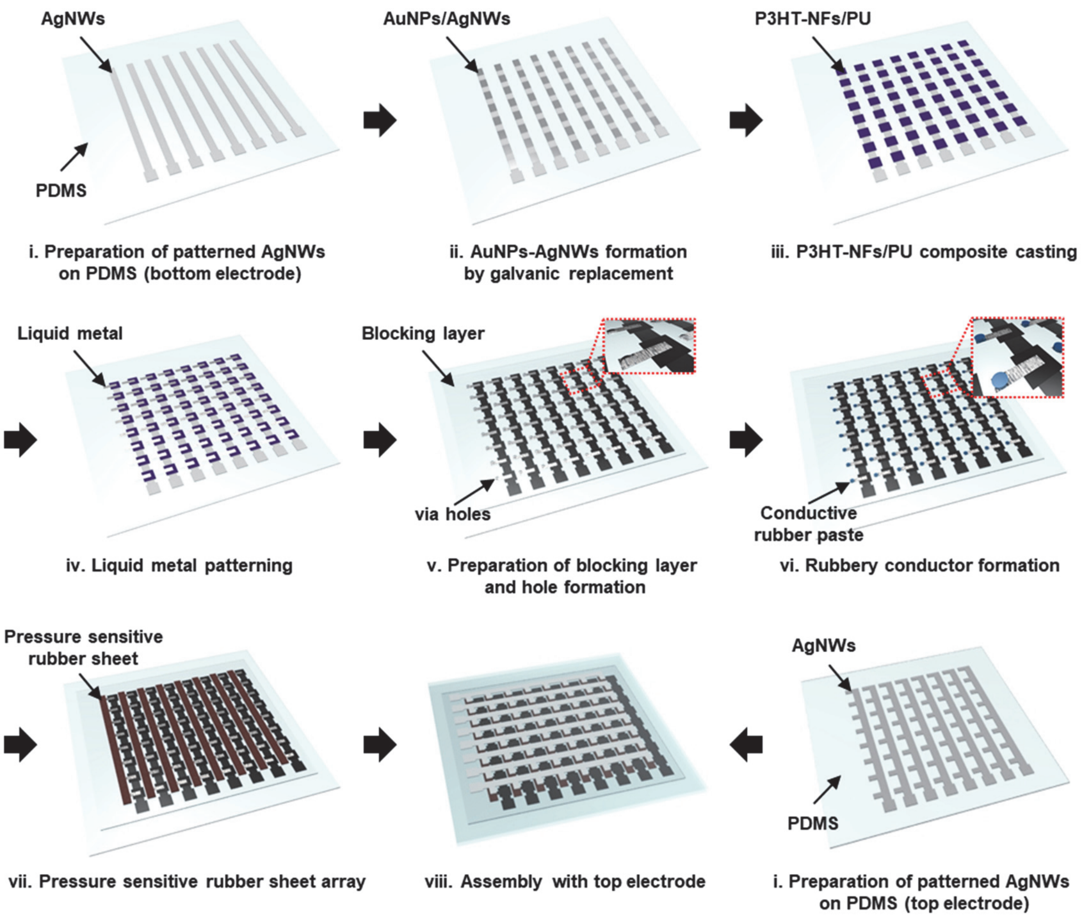

Fig. S11. Schematic illustration of the fabrication process of the fully rubbery multiplexed tactile sensor array.

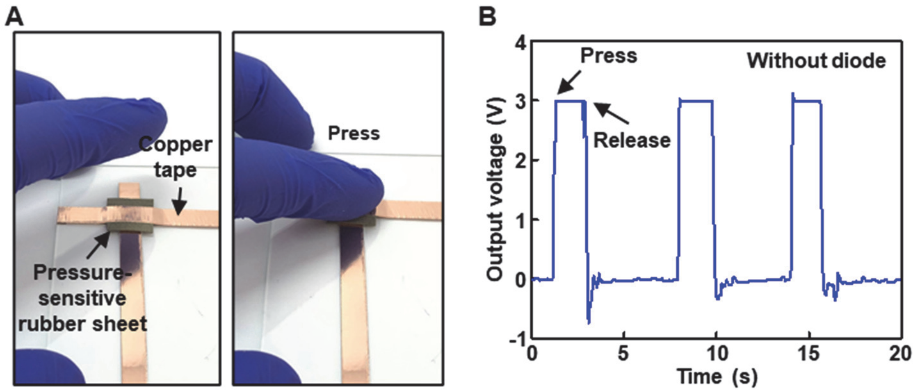

Fig. S12. Tactile sensing test of the pressure-sensitive rubber sheet. (**A**) Optical images of pressing the pressure-sensitive rubber sheet. (**B**) Dynamic output voltage response with press or release under 3 V applied.

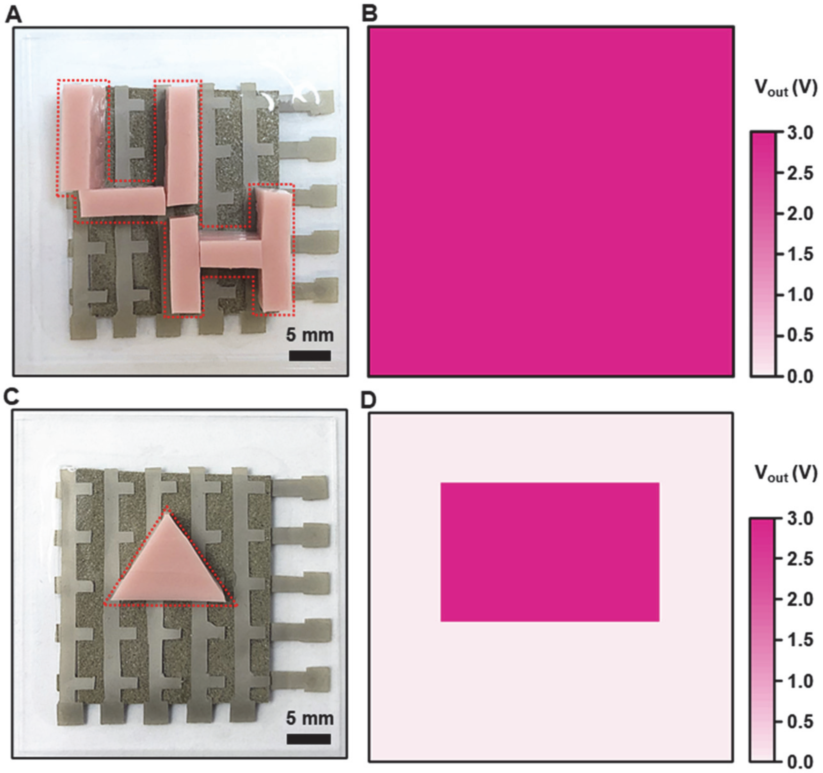

Fig. S13. Cross-talk effect of the rubbery tactile sensor array without multiplexing. (**A**) An Optical image of the tactile sensor array with a letter-shaped object (UH) placed on it. (**B**) Output voltage mapping of the tactile sensor array corresponding to (A). (**C**) An Optical image of the tactile sensor array with a triangle-shaped placed on it. (**D**) Output voltage mapping of the tactile sensor array corresponding to (C).

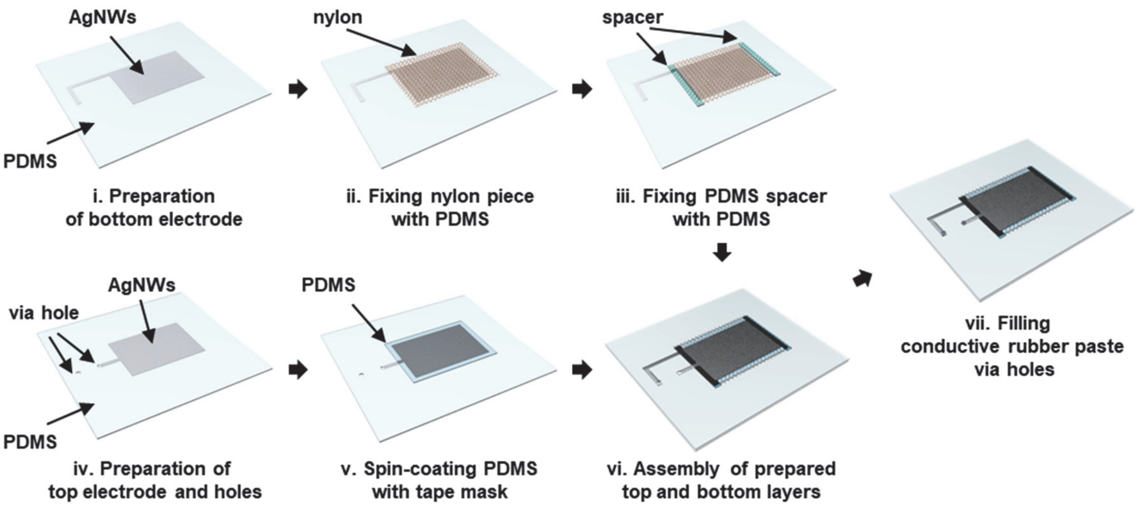

Fig. S14. Schematic illustration of the fabrication process of the rubbery TENG.

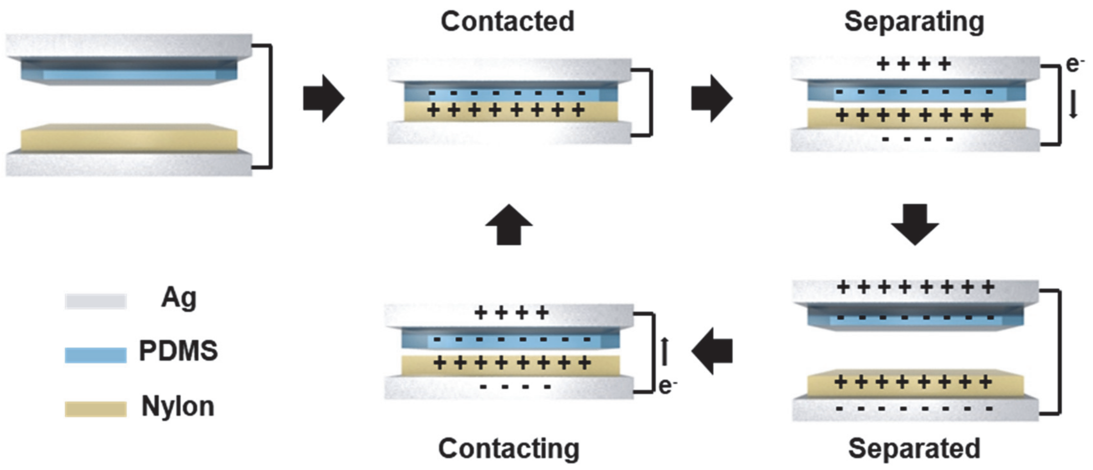

Fig. S15. Working mechanism of the rubbery TENG in contact-separation mode.

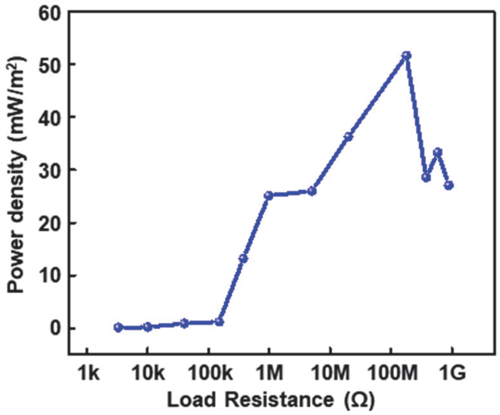

Fig. S16. Dependence of the power density of the rubbery TENG on the external resistors.

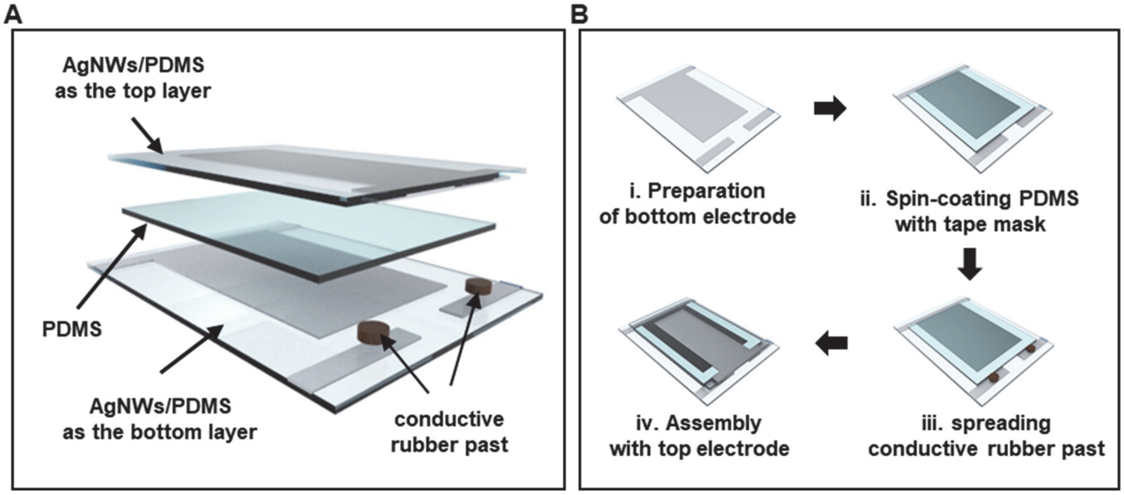

Fig. S17. PDMS based rubbery capacitor. (**A**) Schematic illustration in an exploded view of the rubbery capacitor. (**B**) A fabrication process of the rubbery capacitor.

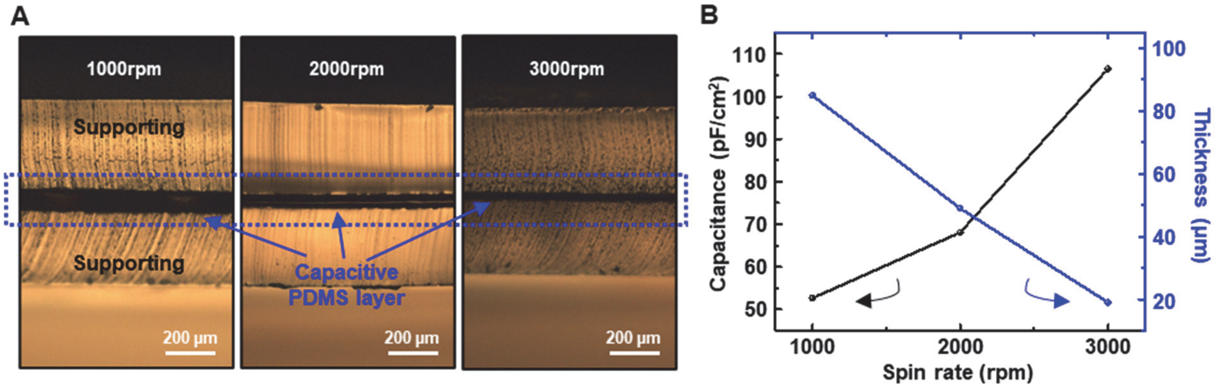

Fig. S18. Rubbery capacitors. (**A**) Cross-sectional optical images of the rubbery capacitors. (**B**) Capacitances of the rubbery capacitors with different thicknesses.

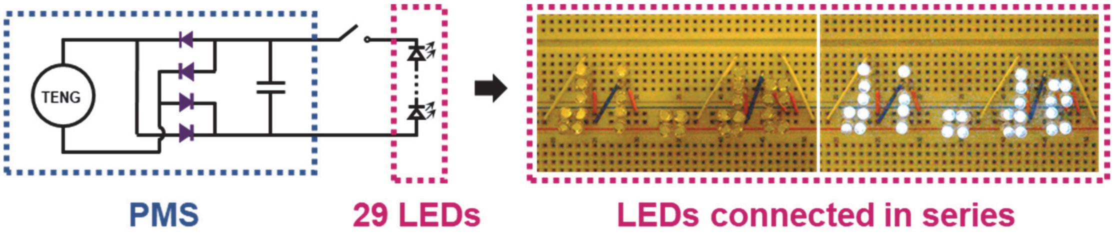

Fig. S19. Schematic diagram of the connection between PMS and 29 LEDs array.

REFERENCES AND NOTES

1. E. Hara, A high power symmetrical cockcroft-Walton type voltage multiplier circuit using silicon diodes. *Nucl. Instrum. Methods* **54**, 91–97 (1967).

2. J. C. Salmon, Operating a three-phase diode rectifier with a low-input current distortion using a series-connected dual boost converter. *IEEE Trans. Power Electron.* **11**, 592–603 (1996).

3. Q. Luo, Y. Cheng, J. Yang, R. Cao, H. Ma, Y. Yang, R. Huang, W. Wei, Y. Zheng, T. Gong, J. Yu, X. Xu, P. Yuan, X. Li, L. Tai, H. Yu, D. Shang, Q. Liu, B. Yu, Q. Ren, H. Lv, M. Liu, A highly CMOS compatible hafnia-based ferroelectric diode. *Nat. Commun.* **11**, 1391 (2020).

4. C. K. Jeong, J. Lee, S. Han, J. Ryu, G. T. Hwang, D. Y. Park, J. H. Park, S. S. Lee, M. Byun, S. H. Ko, A hyper-stretchable elastic-composite energy harvester. *Adv. Mater.* **27**, 2866–2875 (2015).

5. H. Li, Y. Ding, H. Ha, Y. Shi, L. Peng, X. Zhang, C. J. Ellison, G. Yu, An all-stretchable-component sodium-ion full battery. *Adv. Mater.* **29**, 1700898 (2017).

6. S. K. Karan, S. Maiti, J. H. Lee, Y. K. Mishra, B. B. Khatua, J. K. Kim, Recent advances in self-powered tribo-/piezoelectric energy harvesters: All-in-one package for future smart technologies. *Adv. Funct. Mater.* **30**, 2004446 (2020).

7. B.-Y. Kim, H.-B. Lee, N.-E. Lee, A durable, stretchable, and disposable electrochemical biosensor on three-dimensional micro-patterned stretchable substrate. *Sens. Actuators B Chem.* **283**, 312–320 (2019).

8. T. Ha, J. Tran, S. Liu, H. Jang, H. Jeong, R. Mitbander, H. Huh, Y. Qiu, J. Duong, R. L. Wang, A chest-laminated ultrathin and stretchable E-Tattoo for the measurement of electrocardiogram, seismocardiogram, and cardiac time intervals. *Adv. Sci.* **6**, 1900290 (2019).

9. Z. Yang, R. Huang, B. Zheng, W. Guo, C. Li, W. He, Y. Wei, Y. Du, H. Wang, D. Wu, Highly stretchable, adhesive, biocompatible, and antibacterial hydrogel dressings for wound healing. *Adv. Sci.* **8**, 2003627 (2021).

10. J. A. Rogers, T. Someya, Y. Huang, Materials and mechanics for stretchable electronics. *Science* **327**, 1603–1607 (2010).

11. J. Ausra, S. J. Munger, A. Azami, A. Burton, R. Peralta, J. E. Miller, P. Gutruf, Wireless battery free fully implantable multimodal recording and neuromodulation tools for songbirds. *Nat. Commun.* **12**, 1968 (2021).

12. H. Song, G. Luo, Z. Ji, R. Bo, Z. Xue, D. Yan, F. Zhang, K. Bai, J. Liu, X. Cheng, Highly-integrated, miniaturized, stretchable electronic systems based on stacked multilayer network materials. *Sci. Adv.* **8**, eabm3785 (2022).

13. R. Goldoni, Y. Ozkan-Aydin, Y.-S. Kim, J. Kim, N. Zavanelli, M. Mahmood, B. Liu, F. L. Hammond III, D. I. Goldman, W.-H. Yeo, Stretchable nanocomposite sensors, nanomembrane interconnectors, and wireless electronics toward feedback–loop control of a soft earthworm robot. *ACS Appl. Mater. Interfaces* **12**, 43388–43397 (2020).

14. G. Jin, Y. Sun, J. Geng, X. Yuan, T. Chen, H. Liu, F. Wang, L. Sun, Bioinspired soft caterpillar robot with ultra-stretchable bionic sensors based on functional liquid metal. *Nano Energy* **84**, 105896 (2021).

15. Z. Ye, G. Pang, K. Xu, Z. Hou, H. Lv, Y. Shen, G. Yang, Soft robot skin with conformal adaptability for on-body tactile perception of collaborative robots. *IEEE Robot. Autom. Lett* **7**, 5127–5134 (2022).

16. Y. Son, J. Li, R. L. Peterson, In situ chemical modification of Schottky barrier in solution-processed zinc tin oxide diode. *ACS Appl. Mater. Interfaces* **8**, 23801–23809 (2016).

17. J. Y. Wu, Y. T. Chun, S. Li, T. Zhang, D. Chu, Electrical rectifying and photosensing property of Schottky diode based on MoS_2. *ACS Appl. Mater. Interfaces* **10**, 24613–24619 (2018).

18. X. Feng, X. Zhao, L. Yang, M. Li, F. Qie, J. Guo, Y. Zhang, T. Li, W. Yuan, Y. Yan, All carbon materials pn diode. *Nat. Commun.* **9**, 3750 (2018).

19. E. K. Bernardin, C. L. Frewin, R. Everly, J. Ul Hassan, S. E. Saddow, Demonstration of a robust all-silicon-carbide intracortical neural interface. *Micromachines (Basel)* **9**, 451 (2018).

20. T. Widlund, S. Yang, Y.-Y. Hsu, N. Lu, Stretchability and compliance of freestanding serpentine-shaped ribbons. *Int. J. Solids Struct.* **51**, 4026–4037 (2014).

21. P. Won, J. J. Park, T. Lee, I. Ha, S. Han, M. Choi, J. Lee, S. Hong, K. J. Cho, S. H. Ko, Stretchable and transparent kirigami conductor of nanowire percolation network for electronic skin applications. *Nano Lett.* **19**, 6087–6096 (2019).

22. N. Matsuhisa, S. Niu, S. J. K. O'Neill, J. Kang, Y. Ochiai, T. Katsumata, H.-C. Wu, M. Ashizawa, G.-J. N. Wang, D. Zhong, X. Wang, X. Gong, R. Ning, H. Gong, I. You, Y. Zheng, Z. Zhang, J. B. H. Tok, X. Chen, Z. Bao, High-frequency and intrinsically stretchable polymer diodes. *Nature* **600**, 246–252 (2021).

23. P. Li, Z.-H. Lu, Interface engineering in organic electronics: Energy-level alignment and charge transport. *Small Science* **1**, 2000015 (2020).

24. M. Gobbi, L. Pietrobon, A. Atxabal, A. Bedoya-Pinto, X. Sun, F. Golmar, R. Llopis, F. Casanova, L. E. Hueso, Determination of energy level alignment at metal/molecule interfaces by in-device electrical spectroscopy. *Nat. Commun.* **5**, 4161 (2014).

25. W. Wu, Stretchable electronics: Functional materials, fabrication strategies and applications. *Sci. Technol. Adv. Mater.* **20**, 187–224 (2019).

26. K. Sim, Z. Rao, H. J. Kim, A. Thukral, H. Shim, C. Yu, Fully rubbery integrated electronics from high effective mobility intrinsically stretchable semiconductors. *Sci. Adv.* **5**, eaav5749 (2019).

27. H.-L. Yip, A. K. Y. Jen, Recent advances in solution-processed interfacial materials for efficient and stable polymer solar cells. *Energ. Environ. Sci.* **5**, 5994 (2012).

28. H. Lee, D. Lee, Y. Ahn, E. W. Lee, L. S. Park, Y. Lee, Highly efficient and low voltage silver nanowire-based OLEDs employing a n-type hole injection layer. *Nanoscale* **6**, 8565–8570 (2014).

29. F. Ongul, Solution-processed inverted organic solar cell using V_2O_5 hole transport layer and vacuum free EGaIn anode. *Opt. Mater.* **50**, 244–249 (2015).

30. Y. Sun, Y. Xia, Mechanistic study on the replacement reaction between silver nanostructures and chloroauric acid in aqueous medium. *J. Am. Chem. Soc.* **126**, 3892–3901 (2004).

31. Y. Sun, Y. Wang, Monitoring of galvanic replacement reaction between silver nanowires and $HAuCl_4$ by in situ transmission X-ray microscopy. *Nano Lett.* **11**, 4386–4392 (2011).

32. I. H. Campbell, D. L. Smith, Schottky energy barriers and charge injection in metal/Alq/metal structures. *Appl. Phys. Lett.* **74**, 561–563 (1999).

33. A. Takshi, M. Mohammadi, J. D. Madden, Study the effect of distribution of density of states on the depletion width of organic Schottky contacts. *Solid State Electron.* **52**, 1717–1721 (2008).

34. L. G. de Carli, Y. Juppa, A. J. Cardoso, C. Galup-Montoro, M. C. Schneider, Maximizing the power conversion efficiency of ultra-low-voltage CMOS multi-stage rectifiers. *IEEE Trans. Circuits Syst. I Regul. Pap.* **62**, 967–975 (2015).

35. M. Abolhassani, Modular multipulse rectifier transformers in symmetrical cascaded H-bridge medium voltage drives. *IEEE Trans. Power Electron.* **27**, 698–705 (2012).

36. S. Iqbal, A hybrid symmetrical voltage multiplier. *IEEE Trans. Power Electron.* **29**, 6–12 (2014).

37. Y. Zi, J. Wang, S. Wang, S. Li, Z. Wen, H. Guo, Z. L. Wang, Effective energy storage from a triboelectric nanogenerator. *Nat. Commun.* **7**, 10987 (2016).

38. Y. Song, J. Min, Y. Yu, H. Wang, Y. Yang, H. Zhang, W. Gao, Wireless battery-free wearable sweat sensor powered by human motion. *Sci. Adv.* **6**, eaay9842 (2020).

39. I. Grout, *Digital Systems Design with FPGAs and CPLDs* (Elsevier, 2011).

40. K. Sim, S. Chen, Z. Li, Z. Rao, J. Liu, Y. Lu, S. Jang, F. Ershad, J. Chen, J. Xiao, C. Yu, Three-dimensional curvy electronics created using conformal additive stamp printing. *Nat. Electron.* **2**, 471–479 (2019).

41. B. Shi, Z. Li, Y. Fan, Implantable energy-harvesting devices. *Adv. Mater.* **30**, 1801511 (2018).

42. Z. Liu, H. Li, B. Shi, Y. Fan, Z. L. Wang, Z. Li, Wearable and implantable triboelectric nanogenerators. *Adv. Funct. Mater.* **29**, 1808820 (2019).

43. H. Ouyang, Z. Liu, N. Li, B. Shi, Y. Zou, F. Xie, Y. Ma, Z. Li, H. Li, Q. Zheng, X. Qu, Y. Fan, Z. L. Wang, H. Zhang, Z. Li, Symbiotic cardiac pacemaker. *Nat. Commun.* **10**, 1821 (2019).

44. Q. Zheng, Y. Zou, Y. Zhang, Z. Liu, B. Shi, X. Wang, Y. Jin, H. Ouyang, Z. Li, Z. L. Wang, Biodegradable triboelectric nanogenerator as a life-time designed implantable power source. *Sci. Adv.* **2**, e1501478 (2016).

45. H. Zou, Y. Zhang, L. Guo, P. Wang, X. He, G. Dai, H. Zheng, C. Chen, A. C. Wang, C. Xu, Quantifying the triboelectric series. *Nat. Commun.* **10**, 1–9 (2019).

46. H.-J. Yoon, H. Ryu, S.-W. Kim, Sustainable powering triboelectric nanogenerators: Approaches and the path towards efficient use. *Nano Energy* **51**, 270–285 (2018).

47. R. Zhang, H. Olin, Material choices for triboelectric nanogenerators: A critical review. *EcoMat* **2**, e12062 (2020).

48. Z. Qin, Y. Yin, W. Zhang, C. Li, K. Pan, Wearable and stretchable triboelectric nanogenerator based on crumpled nanofibrous membranes. *ACS Appl. Mater. Interfaces* **11**, 12452–12459 (2019).

49. Y. Zhang, S. Xu, H. Fu, J. Lee, J. Su, K.-C. Hwang, J. A. Rogers, Y. Huang, Buckling in serpentine microstructures and applications in elastomer-supported ultra-stretchable electronics with high areal coverage. *Soft Matter* **9**, 8062–8070 (2013).

50. Y. Zhang, S. Wang, X. Li, J. A. Fan, S. Xu, Y. M. Song, K. J. Choi, W. H. Yeo, W. Lee, S. N. Nazaar, Experimental and theoretical studies of serpentine microstructures bonded to prestrained elastomers for stretchable electronics. *Adv. Funct. Mater.* **24**, 2028–2037 (2014).

51. Z. Xue, H. Song, J. A. Rogers, Y. Zhang, Y. Huang, Mechanically-guided structural designs in stretchable inorganic electronics. *Adv. Mater.* **32**, 1902254 (2020).

52. Y. Lee, J. W. Chung, G. H. Lee, H. Kang, J.-Y. Kim, C. Bae, H. Yoo, S. Jeong, H. Cho, S.-G. Kang, J. Y. Jung, D.-W. Lee, S. Gam, S. G. Hahm, Y. Kuzumoto, S. J. Kim, Z. Bao, Y. Hong, Y. Yun, S. Kim, Standalone real-time health monitoring patch based on a stretchable organic optoelectronic system. *Sci. Adv.* **7**, eabg9180 (2021).

53. B. Ying, R. Z. Chen, R. Zuo, J. Li, X. Liu, An anti-freezing, ambient-stable and highly stretchable ionic skin with strong surface adhesion for wearable sensing and soft robotics. *Adv. Funct. Mater.* **31**, 2104665 (2021).

54. S. Gandla, H. Chae, H.-J. Kwon, Y. Won, H. Park, S. Lee, J. Song, S. Baek, Y.-D. Hong, D. Kim, Ultrafast prototyping of large-area stretchable electronic systems by laser ablation technique for controllable robotic arm operations. *IEEE Trans. Ind. Electron.* **69**, 4245–4253 (2022).

55. H. J. Kim, K. Sim, A. Thukral, C. Yu, Rubbery electronics and sensors from intrinsically stretchable elastomeric composites of semiconductors and conductors. *Sci. Adv.* **3**, e1701114 (2017).

56. J. Y. Oh, M. Shin, T. I. Lee, W. S. Jang, Y. Min, J.-M. Myoung, H. K. Baik, U. Jeong, Self-seeded growth of poly(3-hexylthiophene) (P3HT) nanofibrils by a cycle of cooling and heating in solutions. *Macromolecules* **45**, 7504–7513 (2012).

57. J. Ji, S. Ma, F. Shan, F. Wang, Y. Song, Improving the performance of ternary bulk heterojunction polymer cell by regioregular poly (3-hexylthiophene)-grafted oxide graphene on in situ doping of CdS. *J. Mater. Sci.* **51**, 7395–7406 (2016).

58. R. C. M. Dias, A. M. Góes, R. Serakides, E. Ayres, R. L. Oréfice, Porous biodegradable polyurethane nanocomposites: Preparation, characterization, and biocompatibility tests. *Mater. Res.* **13**, 211–218 (2010).

59. G.-J. A. H. Wetzelaer, P. W. M. Blom, Diffusion-driven currents in organic-semiconductor diodes. *NPG Asia Mater.* **6**, e110 (2014).

60. C. Hyun Kim, O. Yaghmazadeh, Y. Bonnassieux, G. Horowitz, Modeling the low-voltage regime of organic diodes: Origin of the ideality factor. *J. Appl. Phys.* **110**, 093722 (2011).
